# Complex multifractal nature in *Mycobacterium tuberculosis* genome

**DOI:** 10.1038/srep46395

**Published:** 2017-04-25

**Authors:** Saurav Mandal, Tanmoy Roychowdhury, Keilash Chirom, Alok Bhattacharya, R. K. Brojen Singh

**Affiliations:** 1 School of Computational and Integrative Sciences, Jawaharlal Nehru University, New Delhi-110067, India; 2 Department of Health Sciences Research, Mayo Clinic, Rochester, MN, USA; 3 School of Life Sciences, Jawaharlal Nehru University, New Delhi-110067, India

## Abstract

The mutifractal and long range correlation (*C(r*)) properties of strings, such as nucleotide sequence can be a useful parameter for identification of underlying patterns and variations. In this study *C(r*) and multifractal singularity function *f(α*) have been used to study variations in the genomes of a pathogenic bacteria *Mycobacterium tuberculosis*. Genomic sequences of *M. tuberculosis* isolates displayed significant variations in *C(r*) and *f(α*) reflecting inherent differences in sequences among isolates. *M. tuberculosis* isolates can be categorised into different subgroups based on sensitivity to drugs, these are DS (drug sensitive isolates), MDR (multi-drug resistant isolates) and XDR (extremely drug resistant isolates). *C(r*) follows significantly different scaling rules in different subgroups of isolates, but all the isolates follow one parameter scaling law. The richness in complexity of each subgroup can be quantified by the measures of multifractal parameters displaying a pattern in which XDR isolates have highest value and lowest for drug sensitive isolates. Therefore *C(r*) and multifractal functions can be useful parameters for analysis of genomic sequences.

Genomic alteration through a number of mechanisms (mutation, substitution, duplication, deletion, insertion, and selection etc.) in combination with natural selection provides a basis of evolution. However, evolution does maintain some conserved features that are characteristics of the organisms. The generic features of these conserved properties can be characterized by the scaling laws^1^,^2^ emerging from one dimensional genome sequence. These laws are preserved and inherited in the complex evolutionary process. Scaling law of an observable *y(x*), which manifests preserved properties in the system, can be quantified through scaling functions *F*[*x, y(x*)] and Γ[*y(x*)]^3^,^4^, and follows self-affine process for any scale factor *c*^5^, given by


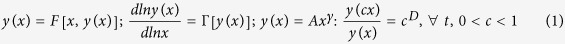


where, *A* is a constant, and *D* is the self-similarity dimension of the self-affine process. If this *y(x*) involves a few number of fractal rules then it obeys Mandelbrot’s classical multifractal rules for self-affine process^6^,


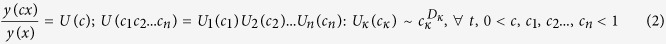


One of the conserved properties is genomic correlation function *C(r*) of the DNA sequence which follows the fractal rule^7^: 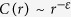, with *D* = *−ε*. The value of *D* is different for different biological processes; for genome length distribution in unicellular organisms *D* = 1/4^8^, for distribution of RNA concentration *D* = 1/4^8^, for metabolic process *D* = −3/4^9^, for heart rate *D* = 1/4^8^, for life span of the organism *D*  =  −1/4^9^, for the distribution of radii of aortas and tree trunks *D* = −3/8^9^.

Multifractal properties of DNA can be characterized by long range correlation maintained in the whole genome^7^, and pseudorandom distribution of nucleotides^10^ following an overall probability distribution. These can be represented as a DNA walk in two dimensional space^10^,^11^. Even though multifractal detrended fluctuations analysis (MF-DFA) technique is particularly important for a varity of time series data analysis^12^, such as sunspot time series^13^, stock exchange rate time series data^14^, complicated earthquake data^15^, social and religious dynamics^16^, traffic flow time series^17^, energy stocks data^18^, brain EEG data^19^, human DNA sequence^20^, the application of this technique for analysis of Next Generation Sequencing (NGS) data of organisms for extraction of useful information may be challenging.

*Mycobacterium tuberculosis* is a slow growing pathogen that causes Tuberculosis (TB) and it is one of the major public health challange particularly among lower and middle income countries^21^,^22^. Drug resistance is one of the major concerns for treatment of patients with this disease and occurrence of extreme drug resistance (XDR) may make the scenario even worse. Drug resistant genes such as *rpoB*^23^,^24^, *inhA*^25^, *katG*^26^, *gyrA*^27^, *ahpC*^28^, *embB*^29^, *pncA*^30^ have been experimentally identified. Different isolates of this bacterium have been classified into various lineages using sequence features and these lineages show correlation with geographical location. Recent genomic studies have found relationship between sequence differences among different isolates (represented by single nucleotide polymorphisms or SNPs and different repetitive sequences) and lineages^31^,^32^. Drug resistance isolates can be classified into different categories depending upon level of resistance. Multi Drug Resistance isolates (MDR) are insensitive to a few drugs whereas XDR isolates are resistant to a number of drugs. In recent times there has been an increase in the number of patients infected with both MDR-TB and XDR-TB, over 480,000 people developed multidrug-resistance TB in 2014 22. India, the Russian Federation and the Peoples Republic of China reported half of the cases of MDR-TB and an estimate of around 9.7% of MDR-TB cases are likely to be also XDR-TB^22^. Changes in genomic sequences are not distributed randomly, some regions (hotspots) display high level of variations whereas a few others are highly conserved (coldspots)^33^. In our analysis we considered a few genes that display significant variations among drug resistant strains and are thought to be involved in drug resistance, such as *rpoB, phoP* and *phoR*. Sequences from some of these genes that map to the same strand of the genome from different *M. tuberculosis* isolates were concatenated to make a single sequence for multifractal analysis^34^. These sequences were obtained from NGS datasets of available isolates^35^,^36^. The results showed that C(r) and Multifractal analysis can be useful parameters for classification of drug resistant isolates.

## Results and Discussion

### Theory of multifractality in genome evolution

**Figure 1:**
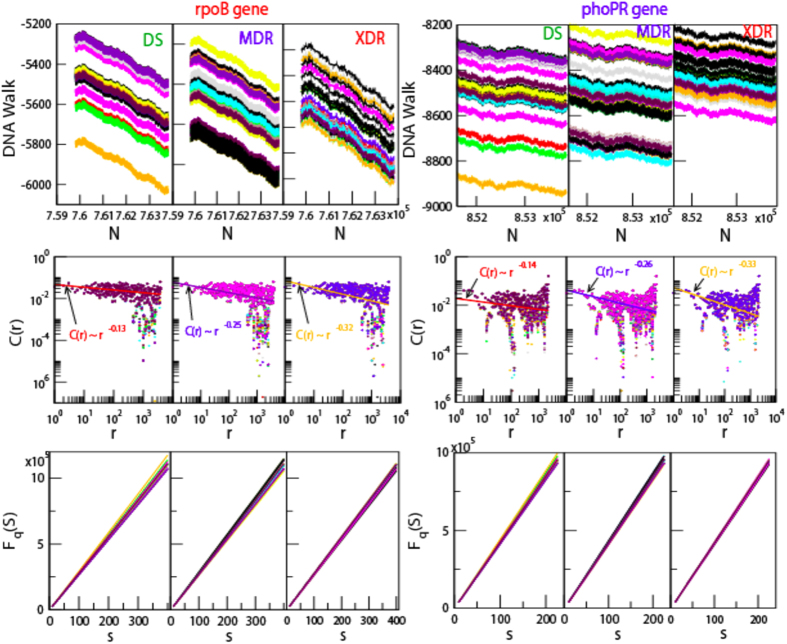
Multifractal and correlation function behaviors of *rpoB* gene (sequence positions: 759807–763325), *phoP* and *phoR* gene combined together(sequence positions: 851608 to 853853) in *M. tuberculosis* genome. (**a**) DNA walks of forty isloates each of DS, MDR and XDR of *M. tuberculosis* (panels of uppermost row). (**b**) Corresponding plots of correlation functions (*C(r*) versus *r*) of the three types of isloates (panels of middle row). Straight lines are power law fits on the data (for *rpoB* gene: DS: 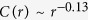; MDR: 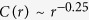; XDR: 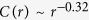 and for *phoPR* gene complex: DS: 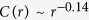; MDR: 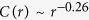; XDR: 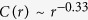). (c) Plots of fluctuation function (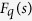) with respect to *s* for the corresponding three types of *M. tuberculosis* isloates showing power law nature (panels of lowermost or third row).

**Figure 2:**
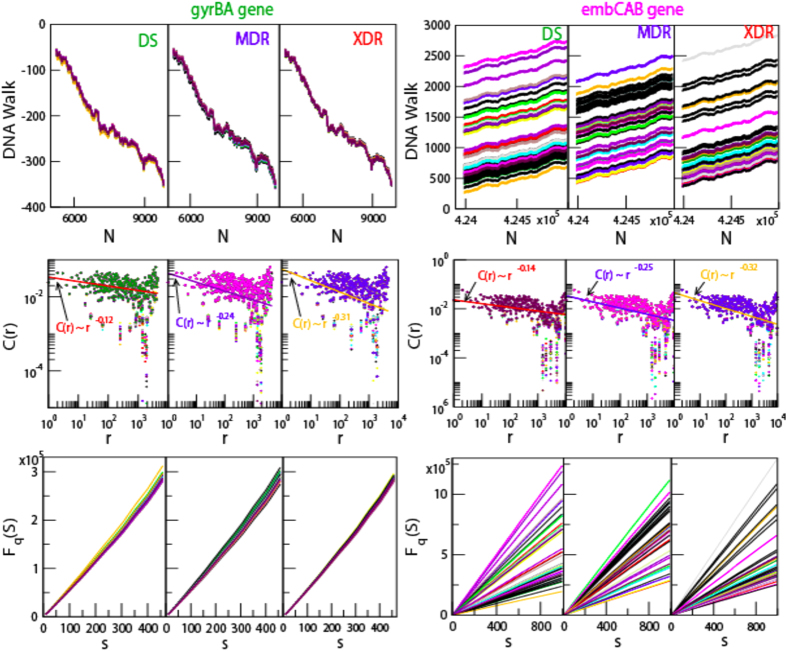
Multifractal and correlation function behaviors of *gyrB* gene and *gyrA* gene concatenated together (sequence positions: 5240–9810) and *embC, embA* and *embB* gene concatenated together with sequence position from 4239863 to 4249810 in *M. tuberculosis* genome. (**a**) DNA walks of forty isloates each of DS, MDR and XDR of *M. tuberculosis* (panels of uppermost row). (**b**) Corresponding plots of correlation functions (*C(r*) versus *r*) of the three types of isloates (panels of middle row). Straight lines are power law fits on the data (for *gyrBA*: DS: 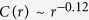; MDR: 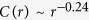; XDR: 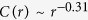 and for *embCAB*: DS: 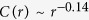; MDR: 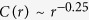; XDR: 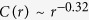). (**c**) Plots of fluctuation function (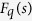) with respect to *s* for the corresponding three types of *M. tuberculosis* isloates showing power law nature (panels of lowermost or third row).

Genome alterations in *M. tuberculosis*, due to various internal and external factors (e.g. continuous encounter with drugs and immune response of the host), is associated with sequence changes that involve substitution with different nucleotide, insertion and deletion, expansion of repeats, recombination and activity of transposable elements. NGS has allowed rapid and inexpensive method of getting complete nucleotide sequence, however the sequences come out as short reads. The nucleotide variation in these isloates are found to be not uniform, and large variations occur in few regions (called *hotspots*)^37^ and few genes only^23^,^24^,^25^,^26^,^27^,^28^,^29^,^30^. One dimensional *DNA walk*^38^ is generated from the genome sequence { *x*_*i*_*; i = 1, 2, …, N*} of each isloate (Fig. 1, Fig. 2 uppermost panels), where *x*_*i*_ = +1 for purine (A and G), and *x*_*i*_ = −1 for pyrimidine (C and T)^10^. Major unaltered portion of the genome of each isolate maintains same long range correlation 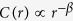 as reference genome with observed root mean square fluctuations of *DNA walk*, 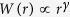, where, *γ* = 1/2 for long range (*r* → *large*), and short range (*r*→0); and *γ* ≠ 1/2 for infinite range *r* → ∞^38^ exhibiting multifractal nature^5^,^6^,^12^. The specific genomic portions of each isolate (concatenated similar drug resistant genes), where significant amount of alterations are exhibited as compared to reference genome, show long range correlations 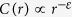 7 (Fig. 1, Fig. 2 middle panels), with fluctuation function *F*_*q*_(*s*) of order *q* (see Methods) obeying power law, 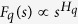 (Fig. 1, Fig. 2 lower most panels), where, *H*_*q*_ is generalized Hurst exponent^12^, showing indication of multifractal nature in the genes.

Since the differences in the phenotypic and genotypic characters of each and every isolates from the reference *M. tuberculosis* genome(*H37Rv*) are due to the variations in the sequences of few hotspots and genes, local scaling properties of *highly polymorphic regions(HPR)* which are concatenated similar drug resistant genes may provide the characteristics of the perturbation induced in the reference genome and gets adapted to it. Consider a *DNA walk* of a *HPR* which can be divided into *m* segments {*u*_*i*_; i = 1, 2, …, N}. Then the probability that the *i*^*th*^ segment having length scale *r* can have *N*_*i*_ observations for large *N*, which is given by 
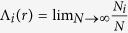
, holds the following power law in the limit *r* → 0^39^,


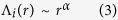


where, *α* is Holder or singularity exponent^40^ which serves as the measure of crowding index in *HPR*. If *N(r, α*) is the number of segments in which Λ_*i*_ has singularity strength between *α* and *α* + Δ*α*, then *N(r, α*) obeys^39^,


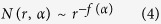


where, *f(α*) is the singularity function which can be related to the observable properties of a certain experimental measure. *f(α*) can also be known as fractal dimension of the set of segments with singularity strength *α*. It can be related to another important generalized dimension *D*_*q*_ of order *q* which can be defined by^41^,^42^,


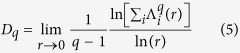


Different values of *D*_*q*_ characterize distribution in the segments with different degree of clustering in it. For non-stationary *DNA walk*, 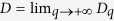 and 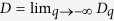 corresponds to fractal dimensions of most and least populated segments respectively. *D*_*q*_ can be related to *f(α*) by employing Legendre transformation to its expression, and can be obtained as^39^,^43^,^44^,


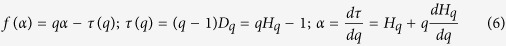


where, *τ* is another classical multifractal scaling exponent^43^,^44^.

For *HPR, f(α*) is singularity spectrum with 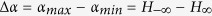 as width of the singularity spectrum, which is a quantitative multifractal strength. Further, *f(α*) → 0, if 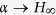 and 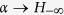
45,46. If the *DNA walk* is monofractal, *H*_*q*_ is independent of *q*, and so from (6), *α* = *constant, τ(q*) is linear function of *q*, and *f(α*) is constant with *α*.

**Figure 3:**
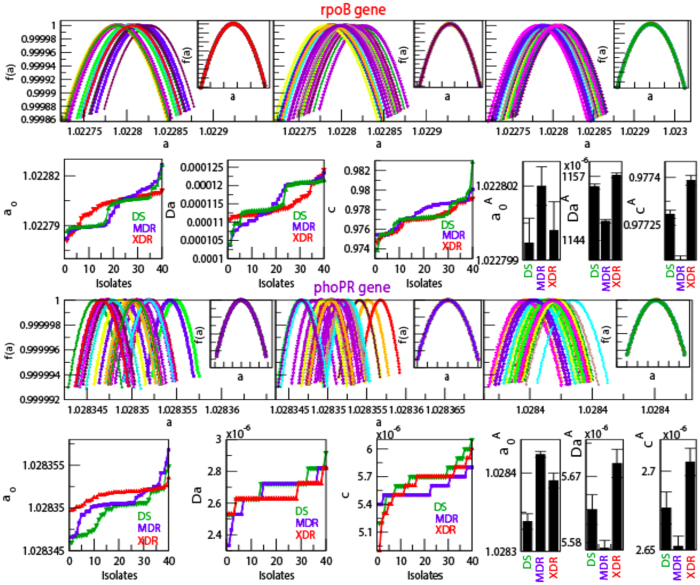
Singularity spectrum of *rpoB* gene and *phoPR* gene complex of *M. tuberculosis* isolates (forty) of each DS, MDR and XDR. (**a**) Plots of singularity function *f(α*) of the three types of isolates with respect to *α* (panels of first row) and their Scaling of *f(α*) by choosing *α*_*c*_ = 1.02275 using interpolation showing self-affine process of the isolates (inside box). (**b**) Properties of multifractal spectral parameters: behaviors of *α*_0_, Δ*α* and *χ* as a function of isolates (colors show types of isolates).

**Figure 4:**
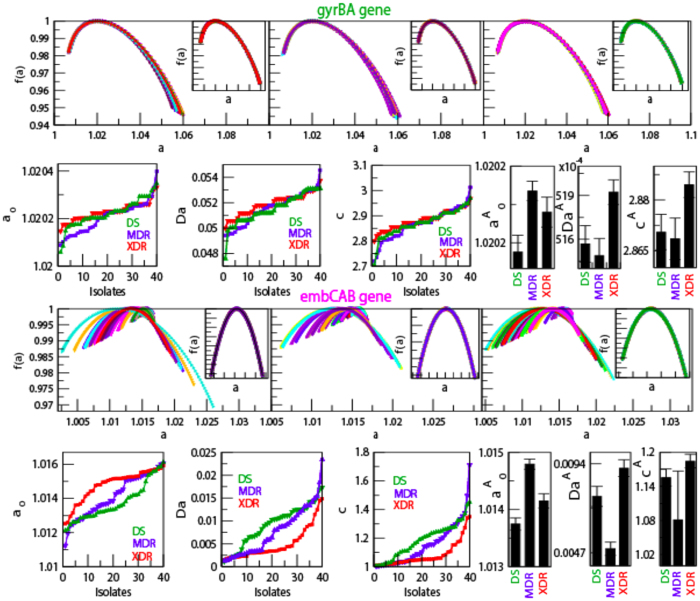
Singularity spectrum of *gyrBA* gene complex(sequence position: 5240 to 9810) and *embCAB* gene complex(sequence position: 4239863 to 4249810) of *M. tuberculosis* isolates (forty) of each DS, MDR and XDR. (**a**) Plots of singularity function *f(α*) of the three types of isolates with respect to *α* (panels of first row) and their Scaling of *f(α*) by choosing *α*_*c*_ = 1.02275 using interpolation showing self-affine process of the isolates (inside box). (**b**) Properties of multifractal spectral parameters: behaviors of *α*_0_, Δ*α* and *χ* as a function of isolates (colors show types of isolates).

The calculated *f(α*) as a function of *α* for forty isolates each of DS, MDR and XDR of *M. tuberculosis* shows different maxima values of *f(α*), but shows similar structural behavior (Figs 3 and 4 upper panels). The average *f(α*) along *α* shows significant difference in three different type of isolates (DS, MDR and XDR), except average *f(α*) values of DS and MDR isolates are approximately overlapping(Figs 3 and 4 the panels in the first and third rows). The scaled behavior of *f(α*) with *α* for each type of isolate shows approximately similar nature (Figs 3 and 4 insets in the panels of first and third rows).

The complexity of the *DNA walk* can be measured by expanding the singularity function *f(α*) around *α*_0_, with *f(α*_0_) → *f*_*max*_ (maximum value of *f(α*)), by Taylor’s series,


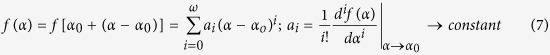


where, *ω* is the degree of the truncated polynomial. Then fitting the *f(α*) data of *DNA walk* with the polynomial (7), the following multifractal parameters can be calculated: *α*_*o*_, *α*_*min*_, *α*_*max*_, and Δ*α* = *α*_*max*_ − *α*_*min*_. The symmetry of each singularity spectrum can be quantified by defining a skew parameter,


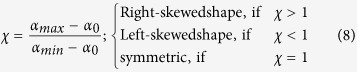


Small value of *α*_0_ correspond to more regular struture in the *HPR*^14^. Δ*α* → *large* indicates stronger multifractality due to richness in structure of the genome. *χ* > 1 reveals the dominance of scaling by small fluctuations and higher Hurst exponents, and indicating the presence of fine structure process in the genome. However, *χ* < 1 indicates the dominance of scaling by large fluctuations of singular spectrum and relatively small Hurst exponents showing correlation in the signal corresponding to absence of fine structure process in the signal. Richness in complexity in the *HPR* corresponds to large value of *α*_0_, wide range of Δ*α*, and *χ* > 1^14^,^47^.

The nature of *α*_0_ for DS and MDR type of isolates are closely similar to each other. This similar behavior is due to the similarity in sequence variation in these two types of isolates, which exhibits similar multifractal behaviors (Figs 3 and 4 extreme left panels in second and fourth rows). The average values of *α*_0_ for the four genes in the three isolates DS, MDR and XDR are found to be different but follow similar behavior (Figs 3 and 4 fourth panels in second and fourth rows). Similar properties of these two types of isolates are also exhibited in the nature of Δ*α* (Figs 3 and 4 second leftmost and fifth panels in second and fourth rows), and in the behaviour of *χ* (Figs 3 and 4 third leftmost and sixth panels in the second and fourth rows). Comparatively large values of Δ*α* and *χ* values in XDR as compared to those of DS and MDR indicates significant richness in multifractality in XDR. Further, since *χ* < 1 (slightly left skewed) for all the three types of isolates, the sequence alteration in the *HPR* is due to genome evolution in *M. tuberculosis*. This induces large fluctuation in the singular function and small in Hurst exponents driving more correlation in the signal and causing destruction of fine structure process in the signal. Since the changes in these parameters are small, these sensitive parameters (*α*_0_, Δ*α* and *χ*) can capture small changes in the multifractal nature due to few sequence alterations in the *HPR* significantly.

### SNP based sequences of *M. tuberculosis* isolates show multifractal nature

The whole genome of each isolate is mapped to the reference genome, and the SNP are arranged in a string without changing their positions but removing the nucleotides in between any pair of SNPs in the genome. The constructed *SNP based sequences* have varied lengths depending on the isolates, ranging from 432 bp to 4000 bp in length. We look at the multifractal properties of these *SNP based sequences* to understand fundamental mechanism of genome evolution (See Table1 in Supplementary file).

**Figure 5:**
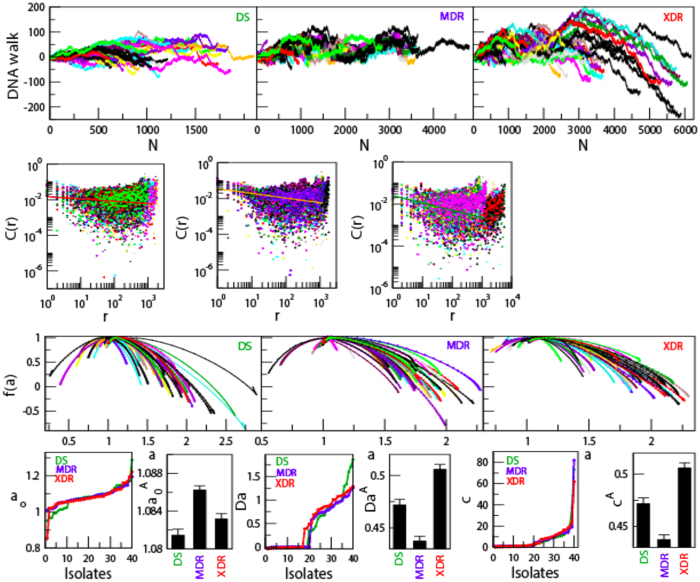
Multifractal and correlation function behaviors of all SNPs (*SNPs based Sequences*) within a genome of forty isolates each from DS, MDR and XDR. (**a**) DNA walks of forty isolates each of DS, MDR and XDR of *M. tuberculosis* (the first three panels of uppermost row). (**b**) Corresponding plots of correlation functions (C(r) versus r) of the three types of isolates (first three panels of second row). Straight lines are power law fits on the data (for DS: 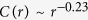; MDR: 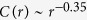; XDR: 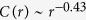). (**c**) Plots of singularity function *f(α*) of the three types of isolates with respect to *α* (first three panels of third row). (**d**) Properties of multifractal spectral parameters: behaviors of *α*_0_, Δ*α* and *χ* as a function of isolates (colors show types of isolates) in the bottom row.

DNA walks of these *SNP based sequences* exhibit different behaviors for the three different classes DS, MDR and XDR (Fig. 5 uppermost row, first three panels). The one dimensional correlation function *C(r*) of these SNP based sequences is calculated using the procedure of Messer *et al*.^48^ (see Methods). The calculated *C(r*)s of all forty isolates of each class are plotted together (Fig. 5 second row), and the data as a whole follows power law,


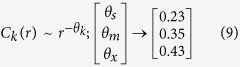


where, *k* indicates isolate types: *k* → *s, m, x* for DS, MDR and XDR respectively. The best fitted curve on the data with power law (9) gives different values of *θ*_*k*_ for different class. This power law behavior of *C(r*) versus *r* for individual as well as groups of isolates (DS, MDR, and XDR) are verified following a standard statistical fitting procedure^49^, and found that the p-values (statistical level of significance) of each fitting on the dataset is found to be more than predicted critical value (*p*−*value* > 0.1). This change in the *θ*_*k*_ could be due to changes in SNPs in the *SNP based sequences* of different types of isolates of *M. tuberculosis*.

The calculated singularity spectra *f(α*) as a function of *α* for various isolates in different types of isolates exhibit different structures (Fig. 5 third row). Calculated *α*_0_ for different types of isolates (Fig. 5 Lowermost row extreme left panel) shows comparatively large values as compared to those of *HPR*, indicating the possibility of associating complex multifractal features in the *SNP based sequences*. The range of singularity spectra Δ*α* for the types of isolates are also significantly large showing wide range of multifractal nature (Fig. 5 Lowermost row third plot). The shape of singularity spectra of all the isolates of different classes are found to be right skewed (*χ* > 1) which are the signature of the existence of fine structures in the *SNP based sequences* due to rich complex multifractal behavior. Further, the values of *α*_0_, Δ*α* and *χ* for XDR *SNP based sequences* are found to be approximately larger than the other types showing richer possession of multifractal properties.

### Scaling in genomic correlation function

**Figure 6:**
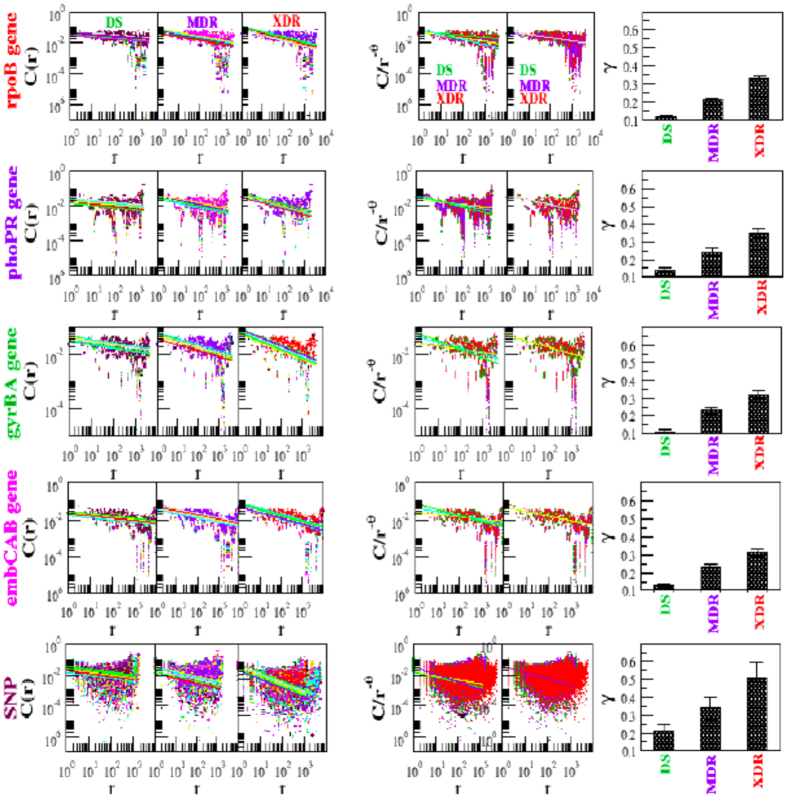
One parameter scaling law in correlation function of *rpoB* gene and SNP based sequences of DS, MDR and XDR of *M. tuberculosis*. (**a**) Scaling in *rpoB* gene for twenty isolates each of DS, MDR and XDR (panels of first row). The straight lines are power law fits to each isolate. The power law exponent (*γ*) for DS, MDR and XDR are given in rightmost panel of second row. The scaled data using Mackinnon and Kramer’s one parameter scaling procedure^4^ (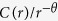 as a function of *r*, see Methods) is shown in first two panels of second row. (**b**) Same scaling procedure is done for SNP based sequences (panels of third and fourth row).

**Figure 7:**
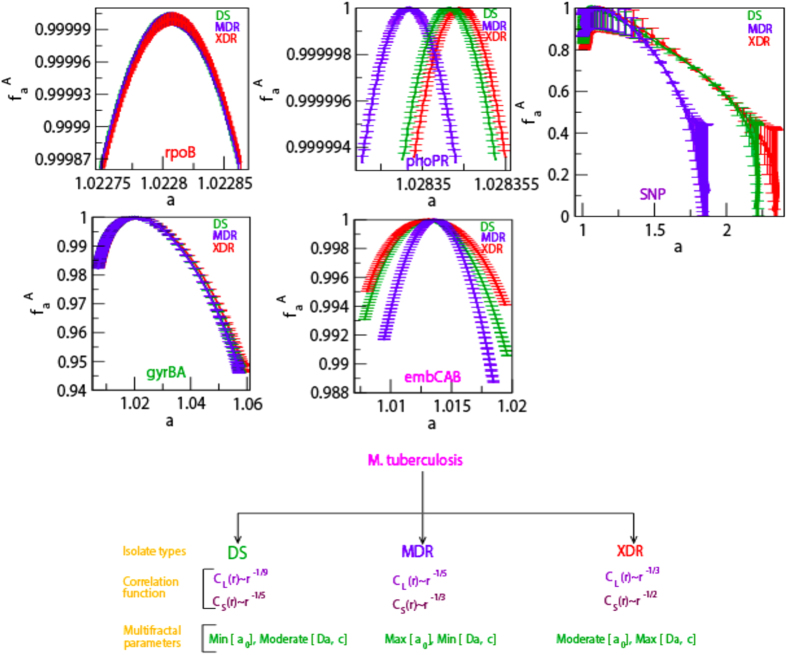
Multifractal and correlation function based classification of DS, MDR and XDR. The average singularity spectra of DS, MDR and XDR of *rpoB, phoPR, gyrBA, embCAB* and SNP based sequences (lower panel) as a function of *α*. The classification of DS, MDR and XDR based on Multifractal parameters and correlation function behaviors.

The changes in *HPR* and *SNP based sequences* in different isolates of DS, MDR and XDR are due to selection of *M. tuberculosis* that are undergone sequence changes allowing resistance to drugs in the course of time^50^. This selection process is the one that allows only some isolates with altered genotypic and phenotypic properties leading to genome evolution^51^,^52^. These changes are species specific and affected very much by many factors including host immune systems and climatic conditions^53^. The spatio-temporal alterations in sequences in the isolates due to sequence alterations (mutations, deletions, duplications, insertions, substitutions, selections) can be nicely modeled using the proposed sequence evolution model^7^,^54^, and some of the observables can be characterized by the dynamics of position dependent one dimensional sequence compositional correlation, 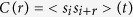. Defining 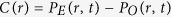, where, *P*_*E*_ and *P*_*E*_ are joint probabilities of finding any two symbols equal and opposite in sign and following their own Master equations, one can arrive at the following evolutionary dynamics of *C(r*) for *r* ≫ 1 (long range correlation),


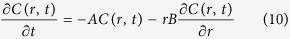


where, *A* and *B* are constants which are functions of the rate constants of sequence alterations. The solution of the equation (10) is given by, 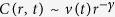; with 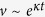, where *κ* is a constant. The stationary (*t* → 0) long range C(*r*) follows power law as we have observed in the *HPR* and *SNP based sequences* (Figs 1, 2, 6 and 7) with 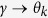. Averaging the values of *θ*_*k*_s of different isolate types in *HPR* and *SNP based sequences* (Fig. 6 Fourth and fifth column panels) respectively, we observe that in long range regime (*r* ≫ 1):

- *HPR*: 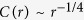; follows 1/4 scaling rule.
- *SNP based sequences*: 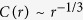; obeys 1/3 scaling law.

However, in short range regime (*r* ≪ 1) the nucleotides in the sequence follow Markov process^7^,^55^, and therefore *C(r*) decays with distance *r* of the nucleotide distribution, 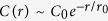, where, *r*_0_ is the characteristics length scale.

The scaling behavior of the *HPR* can be studied by fitting the *C(r*) data of *HPR* of each isolate with equation (9) and analyzing the scaling nature. The fitted lines on the data of DS type (forty isolates) are approximately parallel (Fig. 6 extreme left panel of first row). These data can then be scaled together by using one parameter scaling procedure^4^,^56^ (see Methods) obeying 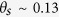 behavior (Fig. 6 fourth and fifth column panels). Applying the same one parameter scaling procedure, data of MDR and XDR isolates can also be scaled obeying 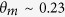 and 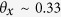 scaling rules respectively (Fig. 6 second, third and fourth column panels). These scaled data of DS, MDR and XDR can then be scaled together (Fig. 6 fourth and fifth column panels) following *θ*~1/4 scaling rule.

The same one parameter scaling procedure can also be done to the *SNP based sequence* data of DS, MDR and XDR isolates (Fig. 6 third and fourth rows). The scaled data follows *θ~*1*/*3 scaling law.

The scaling function Γ can be calculated in this regime using equation (1),


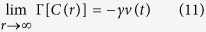


For short range correlated sequences (generated through Markov process), 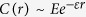
48, and the scaling function can be obtained by,


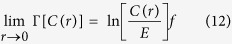


The obeying of one parameter scaling law in NGS genome indicates the signature of self-organization in the system.

### Classification of *M. tuberculosis* isolates

Different isolates(DS, MDR and XDR) can be classified based on the multifractal and correlation properties found in the corresponding *HPR* and *SNP based sequences* (Figs 3, 4, 5, 6 and 7). The average values of singularity spectral parameters of these isolates (Fig. 7) show significant differences: 1. For *α*_0_ (*f(α*_0_) → *constant*) 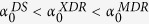; 2. For Δ*α* (measure of multifractal complexity) 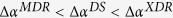, and 3. For *χ* (measure richness in multifractality) 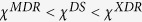. The nature of long range correlation function C(*r*) of these isolate types also exhibit significant behaviors (Fig. 7) as follows,

- For DS: correlation function in *HPR* follows, 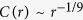 rule; and in *SNP based sequences* obeys 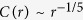.
- For MDR: correlation function in *HPR* follows, 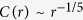 rule; and in *SNP based sequences* obeys 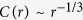.
- For XDR: correlation function in *HPR* follows, 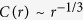 rule; and in *SNP based sequences* obeys 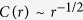.

The behaviors of Multifractal parameters in DS, MDR and XDR of *M. tuberculosis* are found to distinctly different given by:

- For DS: *HPR* and SNP : *Min*[*α*_0_], *Moderate*[Δ*α, χ*].
- For MDR: *HPR* and SNP : *Max*[*α*_0_], *Min*[Δ*α, χ*].
- For XDR: *HPR* and SNP : *Moderate*[*α*_0_], *Max*[Δ*α, χ*].

We classified the NGS sequences of *M. tuberculosis* based on these two distinct properties of Multifractal parameters and correlation function (Fig. 7).

## Conclusion

The genome evolution in *M. tuberculosis* involves alteration of nucleotides in different isolates of DS, MDR and XDR. It is important to remember that genomic alterations continuously takes place and drugs tend to target isolates with appropriate sequence. Normally there are insignificant changes in most of the genome involved in house keeping function needed for the organism to survive and grow^52^. The significant alterations of nucleotides in the genomes of various isolates take place in few regions of the genomes called HPR (hotspots and concatenated genes)^23^,^24^,^25^,^26^,^27^,^28^,^29^,^30^,^34^,^37^. Few of these conserved properties are multifractal nature and correlation function which are being inherited by these isolates from the parent genome with modified rules.

The multifractal nature in the *HPR* of the different *M. tuberculosis* isolates are due to long range correlations with small and large fluctuations, and significant probability distributions in the genome. The singularity spectra of these *HPR* of the isolates is able to capture small range of multifractality from singularity spectral parameters leading to slightly ordered state, but far from monofractality.

The scenario of multifractal properties is quite different in *SNP based sequences* of these isolates which can provide overall properties of the modified genome. These *SNP based sequences* show rich and complex multifractal nature characterized by fine structures in the sequences. This rich multifractal nature in *SNP based sequences* shows the perturbation in the reference genome, with these modified rules (multifractal and correlation nature) within the multifractal boundary for a change for fit survival.

The long range correlation function of *HPR* and *SNP based sequences* of these isolates follow 1/4 and 1/3 scaling rules respectively. The rules in the correlation function may be different in these isolates, but this property is inherited during evolution. Further, the correlation functions in different isolates follow one parameter scaling law indicating that it is one of the properties which keeps genome integrity.

## Methods

### DNA walk of *M. tuberculosis* NGS data

**Figure 8:**
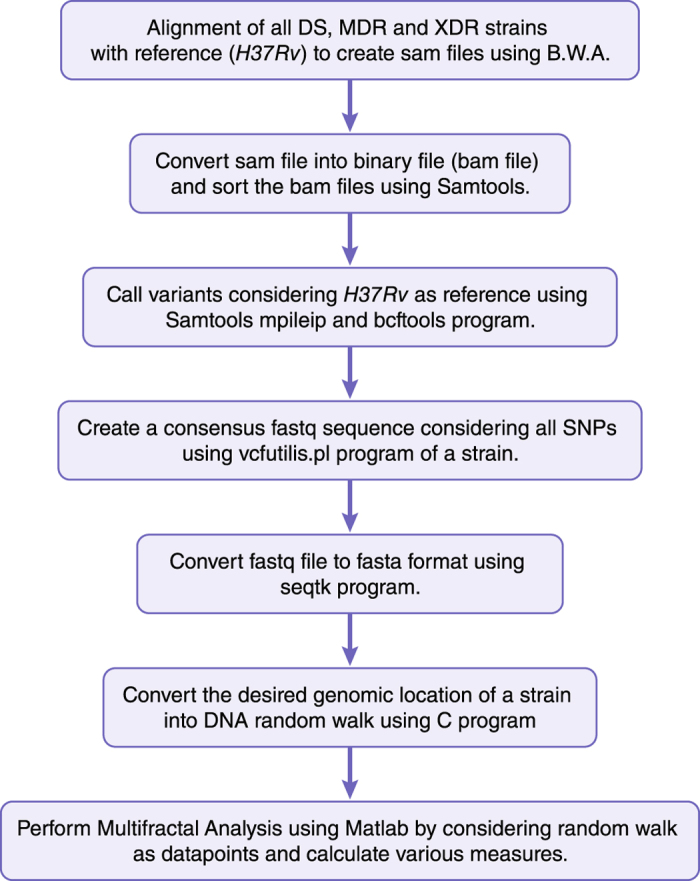
Computational Pipeline for Multifractal Analysis in *M. tuberculosis* bacterium.

The reads of the isolates of *M. tuberculosis* are downloaded from the Sequence Read Archive (SRA)^35^,^36^. Total 120 isolates are considered for our analysis. Forty isolates each from Drug sensitive (DS), Multi Drug Resitant (MDR) and Extremely Drug Resistant isolates (XDR) are considered. The reads are intially mapped to the reference genome *H37Rv* using BWA (Fig. 8)^57^. The BAM file is sorted using samtools and indexing of sorted bam file is performed^58^. In order to create a consensus sequence from the isolates the output of *samtools mpileup* is piped into *bcftools view* command, which in turn is piped into *vcfutilis.pl* program and finally a fastq file is created for the respective isolate. The fastq file is then converted into fasta file using *seqtk* program.

The fasta file is converted in *DNA walk* {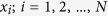} by considering purine (A and G) as step up (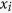 = +1) and pyrimidine (C and T) as step down (*x*_*i*_ = −1)^38^. The *DNA walk* is considered to be a non stationary time series data due to its stochastic behavior which in turn can be used for various properties using Multifractal Detrened Fluctuation Average (MFDFA) analysis using Matlab program^59^,^60^,^61^. The detail procedure is shown in a flowchart below^8^.

### Multifractal DFA approach

Multifractal detrended fluctuation analysis (MF-DFA) is a powerful technique to study fractal properties in nonstationary time series, and associated important correlations charactering the system^39^. Various important parameters which characterize the fractal nature of the time series and related properties, namely, Hurst exponent (*H*), generalized dimension (*D*), singularity spectrum (*f*) etc can be calculated numerically using a method adopted by Kantelhardt *et al*.^12^ as summerized below. Firstly, the time series signal 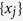 of length *N* is taken as random walk, and can be represented by the profile, 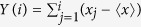, where, 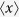 is mean value of the signal, and 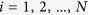. Second, the profile *Y(i*) is now divided into 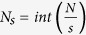 equal nonoverlapping equal segments of size *s*. To take into account all data points, 2*N*_s_ segments are considered by counting starting from both ends of the data. Third, the following variance is determined,


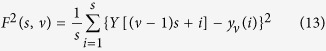


where, 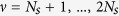, and 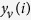 is the fitting polynomial in segment *ν*. Fourth, the qth order fluctuation function is estimated by averaging over all segments,


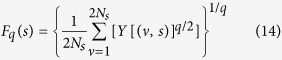


Fifth, the scaling behavior of the function 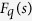 is represented by,


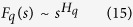


where, *H*_*q*_ is the generalized Hurst exponent, which represents the measure of self-similarity and correlation properties of the signal. Then, *H*_*q*_ is related to classical scaling exponent *τ(q*) as,


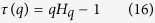


and from the definition of Holder exponent, 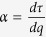, the singularity function 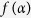
39 is given by,


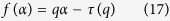


Then, generalized fractal dimension of the signal is measured by,


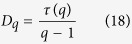


Now, *D*_0_, for *q* = 0, is the fractal or Hausdorff dimension, *D*_1_ is information dimension and *D*_2_ represents correlation dimension^39^. Multifractal signature in the time series can be observed in the system if there exists significant dependence of *H*_*q*_ on *q* in the time series due to different scaling nature of small and large fluctuations^12^. Positive dependence of *H*_*q*_ on *q* indicates the scaling behavior of the time series segments with large fluctuations, whereas negative dependence of *H*_*q*_ on *q* exhibits scaling behavior in the time series segments with small fluctuations. Further, in multifractal time series, small and large fluctuations are characterized by large and small values of 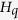.

### Procedure for generating correlation function data

Correlation function 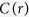 of one dimensional genomic sequence 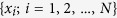 of length 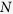 can be calculated following Messer *et al*. procedure^7^ defined by,


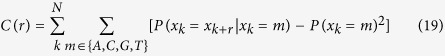


where, 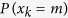 is the probability of finding a base *m* at position *k* in the genomic sequence, and 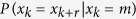 is the conditional probability to find the same base *m* at a distance *r* from *k*.

### One parameter scaling law in correlation function

The calculated correlation function C(*r*) of *HPR* of different isolates of NGS data of *M. tuberculosis*, where significant variation of sequences take place (hotspots and genes), follow power law behavior with approximately parallel fitted lines on *HPR* of different isolates (Fig. 2). This power law fitting on the data is verified and confirmed by following the fitting procedure proposed by Clauset *et al*.^62^, where the value of *p* (statistical significant level) of each fitting is found to be larger than 0.1 which is the critical value of verifying that each data follow power law. We then follow one parameter scaling theory^3^,^4^,^56^ to scale the data given by


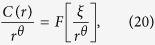


where *F* is a scaling function. The form of the scaling function *F* and values of scaling exponent *θ* for *DS, MDR* and *XDR* isolates can be obtained by scaling the data of these isolates by fitting on the scaled data. Following this scaling procedure, and with the choice of *ξ*, we found that *F* → *constant* and obtained the following scaling law:


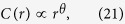


where 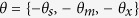 for *HPR* and *SNP based sequence* of different isolates of *DS, MDR* and *XDR* respectively.

### Datasets

NGS datasets were downloaded from European Nucleotide Archive(ENA), EMBL. In Supplementary file Accession numbers and isolate Names are mentioned. Some SNP sequences were downloaded from Genome-based Mycobacterium Tuberculosis Variation (GMTV) Database.

## Additional Information

**How to cite this article:** Mandal, S. *et al*. Complex multifractal nature in *Mycobacterium tuberculosis* genome. *Sci. Rep.* **7**, 46395; doi: 10.1038/srep46395 (2017).

**Publisher's note:** Springer Nature remains neutral with regard to jurisdictional claims in published maps and institutional affiliations.

## Supplementary Material

Supplementary File
