# Supplementary material for: Complex multifractal nature in Mycobacterium tuberculosis genome
Source: Sci Rep. 2017 Apr 25;7:46395. doi: 10.1038/srep46395 (PMC5404331; doi:10.1038/srep46395)
Supplement: Supplementary File [file srep46395-s1.pdf]

Complex multifractal nature in *Mycobacterium tuberculosis* genome.

Saurav Mandal[1],Tanmoy Roychowdhury[3],Keilash Chirom[1],Alok Bhattacharya[1,2], and R.K. Brojen Singh[1*]
[1]*School of Computational and Integrative Sciences,*
Jawaharlal Nehru University, New Delhi-110067, India.
[2]*School of Life Sciences, Jawaharlal Nehru University, New Delhi-110067, India.*
[3]*Department of Health Sciences Research, Mayo Clinic, Rochester, MN, USA.*

TABLE I: **Drug Sensitive(DS)** ***M.tuberculosis*** **isolates considered for Multifractal Analysis**

**S.No.**	**Accession Number**	**Strain Name**
1	*SRR*671723_1.*fastq.gz*, *SRR*671723_2.*fastq.gz*	L2_DS_B-160
2	*SRR*671729_1.*fastq.gz*, *SRR*671729_2.*fastq.gz*	L2_DS_D-xz06169
3	*SRR*671730_1.*fastq.gz*, *SRR*671730_2.*fastq.gz*	L2_DS_E-xz09109
4	*SRR*671776_1.*fastq.gz*, *SRR*671776_2.*fastq.gz*	L2_DS_42061
5	*SRR*671778_1.*fastq.gz*, *SRR*671778_2.*fastq.gz*	L4_DS_42065
6	*SRR*671786_1.*fastq.gz*, *SRR*671786_2.*fastq.gz*	L2_DS_LN037
7	*SRR*671792_1.*fastq.gz*, *SRR*671792_2.*fastq.gz*	L2_DS_LN151
8	*SRR*671793_1.*fastq.gz*, *SRR*671793_2.*fastq.gz*	L2_DS_ShX477
9	*SRR*671800_1.*fastq.gz*, *SRR*671800_2.*fastq.gz*	L2_DS_LN161
10	*SRR*671802_1.*fastq.gz*, *SRR*671802_2.*fastq.gz*	L2_DS_ShX347
11	*SRR*671805_1.*fastq.gz*, *SRR*671805_2.*fastq.gz*	L2_DS_SH147
12	*SRR*671814_1.*fastq.gz*, *SRR*671814_2.*fastq.gz*	L2_DS_SH298
13	*SRR*671823_1.*fastq.gz*, *SRR*671823_2.*fastq.gz*	L2_DS_SH396
14	*SRR*671827_1.*fastq.gz*, *SRR*671827_2.*fastq.gz*	L2_DS_SH045
15	*SRR*671856_1.*fastq.gz*, *SRR*671856_2.*fastq.gz*	L2_DS_HEN06035
16	*SRR*671858_1.*fastq.gz*, *SRR*671858_2.*fastq.gz*	L2_DS_BJ05010
17	*SRR*671870_1.*fastq.gz*, *SRR*671870_2.*fastq.gz*	L2_DS_NM11008
18	*SRR*671875_1.*fastq.gz*, *SRR*671875_2.*fastq.gz*	L2_DS_NM11010
19	*SRR*671876_1.*fastq.gz*, *SRR*671876_2.*fastq.gz*	L2_DS_NM11014
20	*SRR*671877_1.*fastq.gz*, *SRR*671877_2.*fastq.gz*	L2_DS_NM11011
21	*SRR*671879_1.*fastq.gz*, *SRR*671879_2.*fastq.gz*	L2_DS_NM11006
22	*SRR*671874_1.*fastq.gz*, *SRR*671874_2.*fastq.gz*	L4_DS_XJ06090
23	*SRR*671872_1.*fastq.gz*, *SRR*671872_2.*fastq.gz*	L4_DS_XJ06055
24	*SRR*671878_1.*fastq.gz*, *SRR*671878_2.*fastq.gz*	L4_DS_XJ06017
25	*SRR*671846_1.*fastq.gz*, *SRR*671846_2.*fastq.gz*	L4_DS_GZ126
26	*SRR*671773_1.*fastq.gz*, *SRR*671773_2.*fastq.gz*	L4_DS_GZ10145
27	*SRR*671835_1.*fastq.gz*, *SRR*671835_2.*fastq.gz*	L4_DS_GZ10057
28	*SRR*671833_1.*fastq.gz*, *SRR*671833_2.*fastq.gz*	L4_DS_GZ061
29	*SRR*671764_1.*fastq.gz*, *SRR*671764_2.*fastq.gz*	L4_DS_FJ05474
30	*SRR*671832_1.*fastq.gz*, *SRR*671832_2.*fastq.gz*	L4_DS_FJ05395
31	*SRR*671763_1.*fastq.gz*, *SRR*671763_2.*fastq.gz*	L4_DS_FJ05349
32	*SRR*671762_1.*fastq.gz*, *SRR*671762_2.*fastq.gz*	L4_DS_FJ05060
33	*SRR*671860_1.*fastq.gz*, *SRR*671860_2.*fastq.gz*	L4_DS_BJ05043
34	*SRR*671859_1.*fastq.gz*, *SRR*671859_2.*fastq.gz*	L4_DS_BJ05026
35	*SRR*671775_1.*fastq.gz*, *SRR*671775_2.*fastq.gz*	L4_DS_10
36	*SRR*671873_1.*fastq.gz*, *SRR*671873_2.*fastq.gz*	L3_DS_XJ06014
37	*SRR*671741_1.*fastq.gz*, *SRR*671741_2.*fastq.gz*	L3_DS_P-xz09119
38	*SRR*671747_1.*fastq.gz*, *SRR*671747_2.*fastq.gz*	L2_DS_Y-xz09033
39	*SRR*671871_1.*fastq.gz*, *SRR*671871_2.*fastq.gz*	L2_DS_XJ06007
40	*SRR*671803_1.*fastq.gz*, *SRR*671803_2.*fastq.gz*	L2_DS_ShX640

*Electronic address: brojen@jnu.ac.in (Corresponding author)

TABLE II: **Multi Drug Resistant(MDR)** *M.tuberculosis* **Isolates considered for Multifractal Analysis**

**S.No.**	**Accession Number**	**Strain Name**
1	SRR671719_1.fastq.gz, SRR671719_2.fastq.gz	L2_MDR_60
2	SRR671720_1.fastq.gz, SRR671720_2.fastq.gz	L2_MDR_322
3	SRR671721_1.fastq.gz, SRR671721_2.fastq.gz	L2_MDR_142
4	SRR671722_1.fastq.gz, SRR671722_2.fastq.gz	L2_MDR_140
5	SRR671724_1.fastq.gz, SRR671724_2.fastq.gz	L2_MDR_315
6	SRR671727_1.fastq.gz, SRR671727_2.fastq.gz	L2_MDR_A − xz09095
7	SRR671728_1.fastq.gz, SRR671728_2.fastq.gz	L2_MDR_C − xz06202
8	SRR671731_1.fastq.gz, SRR671731_2.fastq.gz	L2_MDR_F − xz09121
9	SRR671733_1.fastq.gz, SRR671733_2.fastq.gz	L2_MDR_H − xz09043
10	SRR671734_1.fastq.gz, SRR671734_2.fastq.gz	L2_MDR_I − xz09061
11	SRR671735_1.fastq.gz, SRR671735_2.fastq.gz	L2_MDR_J − xz09105
12	SRR671736_1.fastq.gz, SRR671736_2.fastq.gz	L2_MDR_L − xz09102
13	SRR671737_1.fastq.gz, SRR671737_2.fastq.gz	L2_MDR_M − xz06013
14	SRR671738_1.fastq.gz, SRR671738_2.fastq.gz	L2_MDR_X − xz09017
15	SRR671739_1.fastq.gz, SRR671739_2.fastq.gz	L2_MDR_N − xz06042
16	SRR671742_1.fastq.gz, SRR671742_2.fastq.gz	L2_MDR_Q − xz09122
17	SRR671744_1.fastq.gz, SRR671744_2.fastq.gz	L2_MDR_S − xz09021
18	SRR671745_1.fastq.gz, SRR671745_2.fastq.gz	L2_MDR_U − xz09006
19	SRR671746_1.fastq.gz, SRR671746_2.fastq.gz	L2_MDR_V − xz09011
20	SRR671869_1.fastq.gz, SRR671869_2.fastq.gz	L2_MDR_144
21	SRR671850_1.fastq.gz, SRR671850_2.fastq.gz	L4_MDR_XZ06055
22	SRR671838_1.fastq.gz, SRR671838_2.fastq.gz	L4_MDR_XZ06030
23	SRR671831_1.fastq.gz, SRR671831_2.fastq.gz	L4_MDR_ShX321
24	SRR671853_1.fastq.gz, SRR671853_2.fastq.gz	L4_MDR_SHX05178
25	SRR671768_1.fastq.gz, SRR671768_2.fastq.gz	L4_MDR_GZ10125
26	SRR671766_1.fastq.gz, SRR671766_2.fastq.gz	L4_MDR_GZ10120
27	SRR671774_1.fastq.gz, SRR671774_2.fastq.gz	L4_MDR_GZ10116
28	SRR671767_1.fastq.gz, SRR671767_2.fastq.gz	L4_MDR_GZ10103
29	SRR671772_1.fastq.gz, SRR671772_2.fastq.gz	L4_MDR_GZ10069
30	SRR671769_1.fastq.gz, SRR671769_2.fastq.gz	L4_MDR_GZ10059
31	SRR671790_1.fastq.gz, SRR671790_2.fastq.gz	L4_MDR_GuangZ29
32	SRR671861_1.fastq.gz, SRR671861_2.fastq.gz	L4_MDR_GuangZ0016
33	SRR671757_1.fastq.gz, SRR671757_2.fastq.gz	L4_MDR_FJ05189
34	SRR671751_1.fastq.gz, SRR671751_2.fastq.gz	L4_MDR_FJ05132
35	SRR671750_1.fastq.gz, SRR671750_2.fastq.gz	L4_MDR_FJ05121
36	SRR671749_1.fastq.gz, SRR671749_2.fastq.gz	L4_MDR_FJ05120
37	SRR671868_1.fastq.gz, SRR671868_2.fastq.gz	L4_MDR_143
38	SRR671840_1.fastq.gz, SRR671840_2.fastq.gz	L2_MDR_XZ06217
39	SRR671851_1.fastq.gz, SRR671851_2.fastq.gz	L2_MDR_XZ06194
40	SRR671839_1.fastq.gz, SRR671839_2.fastq.gz	L2_MDR_XZ06050

TABLE III: **Extremely Drug Resistant(XDR)** *M.tuberculosis* **isolates considered for Multifractal Analysis**

S.No.	Accession Number	Strain Name/Sample ID
1	ERR067578_1.fastq.gz, ERR067578_2.fastq.gz	TB0089
2	ERR067580_1.fastq.gz, ERR067580_2.fastq.gz	TB0091
3	ERR067621_1.fastq.gz, ERR067621_2.fastq.gz	TB0132
4	ERR067637_1.fastq.gz, ERR067637_2.fastq.gz	TB0148
5	ERR067647_1.fastq.gz, ERR067647_2.fastq.gz	TB0158
6	ERR067652_1.fastq.gz, ERR067652_2.fastq.gz	TB0163
7	ERR067661_1.fastq.gz, ERR067661_2.fastq.gz	TB0172
8	ERR067662_1.fastq.gz, ERR067662_2.fastq.gz	TB0173
9	ERR067674_1.fastq.gz, ERR067674_2.fastq.gz	TB0185
10	ERR067743_1.fastq.gz, ERR067743_2.fastq.gz	TB0254
11	ERR067748_1.fastq.gz, ERR067748_2.fastq.gz	TB0259
12	ERR133854_1.fastq.gz, ERR133854_2.fastq.gz	TB0382
13	ERR133906_1.fastq.gz, ERR133906_2.fastq.gz	TB0368
14	ERR133908_1.fastq.gz, ERR133908_2.fastq.gz	TB0478
15	ERR137208_1.fastq.gz, ERR137208_2.fastq.gz	TB0739
16	ERR144600_1.fastq.gz, ERR144600_2.fastq.gz	TB0462
17	ERR158580_1.fastq.gz, ERR158580_2.fastq.gz	TB0733
18	ERR158585_1.fastq.gz, ERR158585_2.fastq.gz	TB0362
19	ERR234560_1.fastq.gz, ERR234560_2.fastq.gz	TB0460
20	ERR234641_1.fastq.gz, ERR234641_2.fastq.gz	TB0369
21	SRR671725_1.fastq.gz, SRR671725_2.fastq.gz	L4_XDR_GuangZ6
22	SRR671726_1.fastq.gz, SRR671726_2.fastq.gz	L2_XDR_GuangZ8
23	SRR671740_1.fastq.gz, SRR671740_2.fastq.gz	L4_XDR_O-xz06008
24	SRR671760_1.fastq.gz, SRR671760_2.fastq.gz	L4_XDR_FJ07028
25	SRR671761_1.fastq.gz, SRR671761_2.fastq.gz	L2_XDR_FJ09143
26	SRR671782_1.fastq.gz, SRR671782_2.fastq.gz	L4_XDR_GuangZ33
27	SRR671783_1.fastq.gz, SRR671783_2.fastq.gz	L2_XDR_GuangZ28
28	SRR671785_1.fastq.gz, SRR671785_2.fastq.gz	L2_XDR_LN081
29	SRR671794_1.fastq.gz, SRR671794_2.fastq.gz	L2_XDR_LN130
30	SRR671799_1.fastq.gz, SRR671799_2.fastq.gz	L2_XDR_LN236
31	SRR671807_1.fastq.gz, SRR671807_2.fastq.gz	L2_XDR_SH170
32	SRR671810_1.fastq.gz, SRR671810_2.fastq.gz	L2_XDR_SH060
33	SRR671811_1.fastq.gz, SRR671811_2.fastq.gz	L2_XDR_SH068
34	SRR671815_1.fastq.gz, SRR671815_2.fastq.gz	L2_XDR_SH311
35	SRR671821_1.fastq.gz, SRR671821_2.fastq.gz	L2_XDR_SH384
36	SRR671842_1.fastq.gz, SRR671842_2.fastq.gz	L2_XDR_FJ07070
37	SRR671862_1.fastq.gz, SRR671862_2.fastq.gz	L4_XDR_GuangZ0026
38	SRR671863_1.fastq.gz, SRR671863_2.fastq.gz	L2_XDR_GuangZ0008
39	SRR671864_1.fastq.gz, SRR671864_2.fastq.gz	L2_XDR_GuangZ0017
40	SRR671865_1.fastq.gz, SRR671865_2.fastq.gz	L2_XDR_293

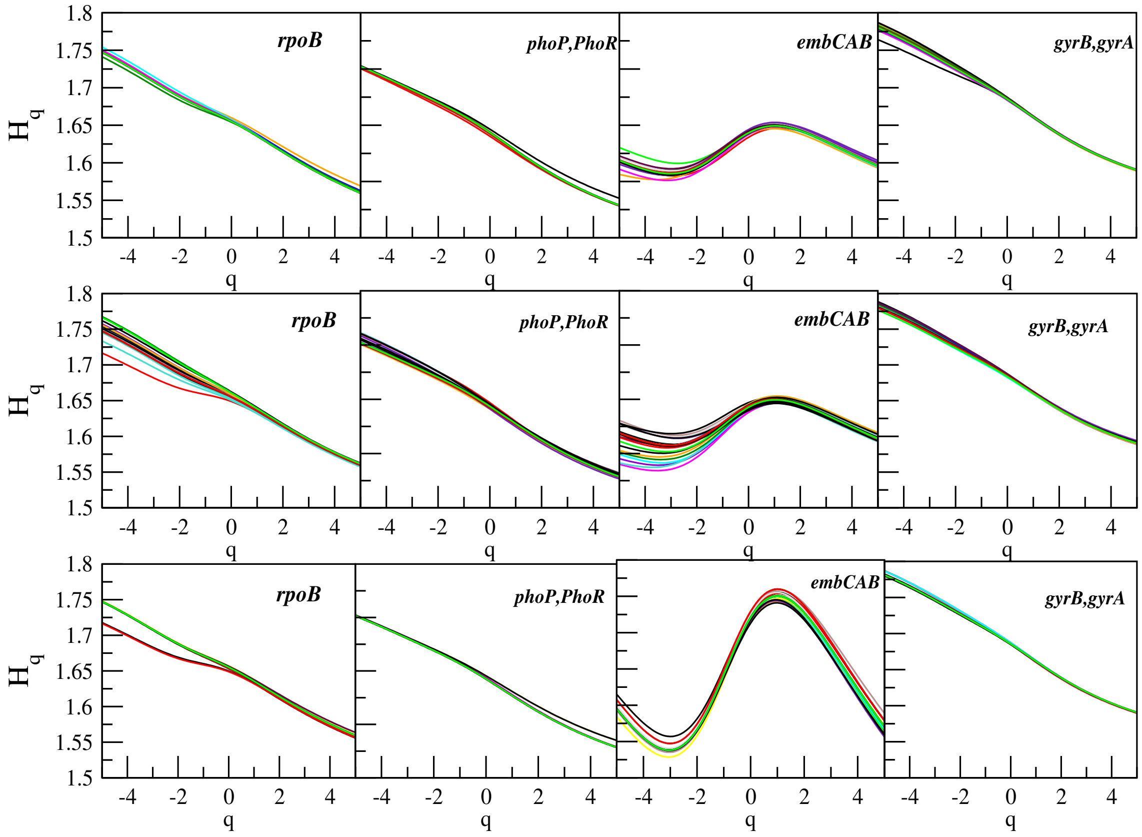

FIG. 1: Plots of Hq vs q of various drug resitant genes. *PhoP* gene(851608 to 852351) and *PhoR* gene(852396 to 853853) were concatenated together to make a single sequence and then performed the analysis. *embC*(4239863 to 4243147),*embA*(4243233 to 4246517) and *embB*(4246514 to 4249810) genes were concatenated as single nucleotide sequence. Similarly *gyrB*(5240 to 7267) and *gyrA*(7302 to 9810) were concatanated. All these were concatenated including the intergenic regions to create a single nucleotide sequence for Multifractal anlaysis.
